# Simulation Analysis of Limit Operating Specifications for Onshore Spoolable Reinforced Thermoplastic Pipes

**DOI:** 10.3390/polym13203480

**Published:** 2021-10-11

**Authors:** Houbu Li, Xuemin Zhang, Haohan Huang, Teng Zhou, Guoquan Qi, Han Ding

**Affiliations:** 1 State Key Laboratory for Performance and Structure Safety of Petroleum Tubular Goods and Equipment Materials, CNPC Tubular Goods Research Institute, Xi’an 710077, China; qiguoquan@cnpc.com.cn (G.Q.); dinghan@cnpc.com.cn (H.D.); 2 School of Materials Science and Engineering, Chang’an University, Xi’an 710064, China; xueminzhang@chd.edu.cn (X.Z.); hhh18438603160@163.com (H.H.); zhoutengaa@yeah.net (T.Z.)

**Keywords:** reinforced thermoplastic pipe, onshore, limit operating specification, finite element method

## Abstract

Spoolable reinforced plastic line pipes (RTPs), exhibiting a series of advantages such as good flexibility, few joints, long single length, light weight, easy installation, etc., have been widely used in the onshore oil and gas industry such as oil and gas gathering and transportation, high pressure alcohol injection, water injection, sewage treatment, and other fields. However, due to the lack of clear standard specificationof the limit operating properties for RTPs, three typical failure modes, i.e., tensile, flexure, and torsion, frequently occur in terrain changes, construction operation, and subsequent application, which seriously affects the promotion and use of RTPs. In this paper, the stress distribution of a non-bonded polyester fiber reinforced high-density polyethylene (HDPE) pipe (DN 150, PN 2.5 MPa) was systematically studied by the finite element method (FEM), and then the limit operating values under the axial tensile, coiled bending, and torsion load were determined. The corresponding experiments were conducted to validate the reliability and accuracy of the FEM model. The FEM results showed that the critical strain for axial tensile was 3%, the minimum respooling bend radius was 1016.286 mm, and the limit torsion angle of this RTP was 58.77°, which are very close to the experimental results. These limit values will be useful to establish normative guidelines for field construction and failure prevention of onshore RTP.

## 1. Introduction

Carbon steels and low alloy steels are the most widely used in ground gathering and transportation system of onshore oilfields. However, in recent years, with the increasingly harsh environment of oilfields, the water content and temperature have gradually increased. On the other hand, the content of corrosive media such as Cl^−^ and CO_2_ has increased, and H_2_S has even appeared in oil and gas wells in some areas, which has brought great risks to the application of steel pipe [1]. Non-metallic and composite pipes have a series of advantages such as corrosion resistance (without internal and external anti-corrosion), smooth inner walls, anti-scaling and waxing, low fluid resistance, anti-wear and anti-erosion, electrical insulation, light weight, easy transportation and construction, and low maintenance costs, etc., whichhave become one of the most important solutions for anti-corrosion of the onshore gathering pipelines [2,3,4,5,6,7].

Currently, the non-metallic and composite pipes used in China’s onshore oilfield systems mainly include glass fiber reinforced thermosettingpipes (GRP), anticorrosion plastic alloy composite pipes, steel skeletonreinforced polyethylene composite pipes, and spoolable reinforced plastic pipes (RTPs) [8]. Among them, RTPs have been widely used in oil and gas fields due to theirgood flexibility, excellent impact resistance, few joints, light weight, low transportation cost, quick and easy installation, etc. Recently, it has becomethe fastest growing non-metallic composite pipe in China. RTPs are mainly used for oil and gas gathering and transportation, high-pressure alcohol injection, oilfield water injection, and sewage treatment, etc. [9]. Since 2011, RTPs have been tested in gas transportation (mixed oil and gas transportation), downhole water injection, and other fields [10,11,12,13,14,15]. Therefore, application of the RTP products in China is developing towards serialization and diversification.

**Figure 1:**
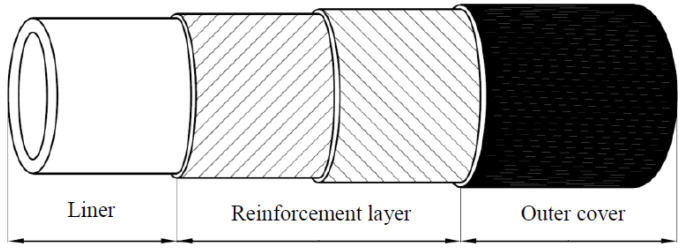
Typical structure of RTP.

RTPs used in onshore oil fields mainly consist of a multi-layer structure: internal thermoplastic liner, reinforcement layer, and outer thermoplastic layer. The typical layer structure of an RTP is shown in Figure 1. For liners, various grades of high-density polyethylene (HDPE) are being used for product temperature sup to 65 °C. If required, RTPs can be designed forhigher operating temperatures by using other appropriate thermoplastic materials such as cross-linked polyethylene (PEX), polyamides (PA), and polyvinylidene fluoride (PVDF). The reinforcement layer is usually made of continuous fiber (or tape) and steel wire (or tape); the fiber materials can be polyester fiber, glass fiber, carbon fiber, and aramid fiber. The outer protective layer is usually high-density polyethylene (HDPE). 

RTPs can be constructedas bonded or non-bonded structures based on the manufacturing process. In bonded construction, the reinforcement fiber tape is fused to the linerand the outer cover to form a solid wall. RTPs withbonded construction offer greater resistance to linercollapse on depressurization or cover blow-off due todiffused gases. However, in non-bonded construction, different layers are mutually independent and relativemovement is allowed.Un-bonded RTPs have the advantages of a simple manufacturing process, high manufacture efficiency, and good flexibility. Therefore, based on consideration of manufacturing efficiency, product cost, and application conditions, the most used RTP in onshore oil fields is the non-bonded polyester fiber reinforced HDPE composite pipe, especially in China.

**Figure 2:**
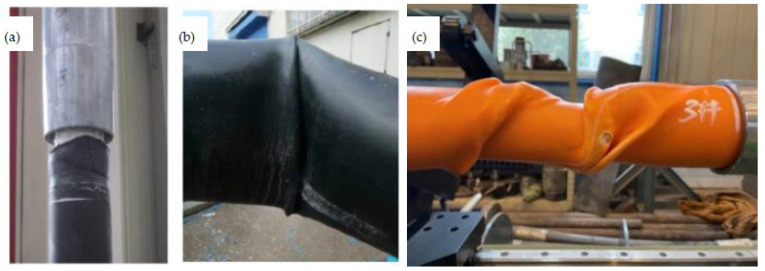
Three typical failure modes of RTP: (**a**) tensile failure; (**b**) buckling failure; (**c**) torsional failure.

As mentioned above, the non-bonded polyester fiber reinforced HDPE composite pipe has been popularized and applied in various fields of onshore oil fields in China. The total use exceeds 40,000 km, and the annual growth rate is higher than 10%. However, with the increasing quantity, three typical failure modes are gradually exposed, as shown in Figure 2, which seriously affect the product promotion and the user’s confidence. One is that the end fittingor pipe body of the RTP failsfrequently due to the tensile overload during the drag construction or subsequent ground subsidence (Figure 2a). The other is buckling failure of the RTP caused by bending overload under the condition of coiling transportation, laying or service with a small bending radius such as sand dunes and hillsides, as shown in Figure 2b.The third is that the pipe body fails due to torsional overload when the RTP rolls along in the process of desert or mountain movement(Figure 2c).

Existing standards and manufacturers have not provided clear specific requirements for the limit operating performance of RTPs such as tensile, bending, and torsion, so there is no useful guideline for oilfield users to effectively control the above failure risks and prevent them.The effective method to determine the limitoperating performance of RTPs is to conduct a large number of full-scale laboratory tests. However, due to a variety of the RTP specifications (diameter range of 50–150 mm, normal pressure range of 1.6–32 MPa), various material types (various thermoplastics and reinforcement materials and types), and different manufacturing processes (number of winding layers, angles, tension, etc.), this method of testing is time-consuming and expensive, and it is difficult to give specific data quickly and accurately.

In order to establish normative guidelines for field construction operation and failure prevention of RTPs, a fast and accurate method to obtain the limit tensile displacement, the minimum respooling bendradius, and the limit torsional radian of the RTP was explored in this paper. Polyester fiber reinforced HDPE pipe, which isthe most widely used RTPin onshore oil fields in China was taken as the research object. A pipe body model of the RTP (DN 150, PN 2.5 MPa) was established by the finite element method, and thereliability of this model was verified by a burst pressure test. On this basis, the stress distributions of the RTP under axial tension, coiled bending, and torsion load were systematically studied by the finite element method. Furthermore, based on the yield strength of the RTP materials such as the thermoplastic liner and reinforced fiber, the limit operating performance of the RTP was calculated and analyzed and further verified by the corresponding experiments.

## 2. Numerical Simulations

**Figure 3:**
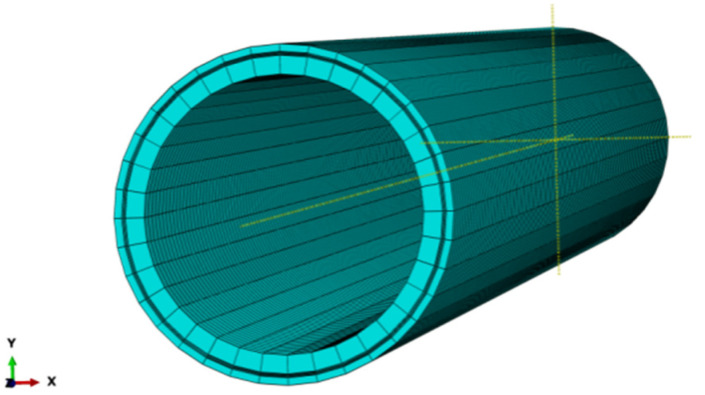
FEM model of RTP.

A finite element (FE) model was established by using ABAQUS software (ABAQUS software Ctd., Providence, RI, USA), as shown in Figure 3. The geometrical parameters of the FE model were in accordance with the ones listed in Table 1. To eliminate the end effect of the pipeline subject to a fixed constraint or connection, a pipe length of 2000 mm was adopted, which is 10 to 20 times of the outside diameter of the RTP (DN 150, PN 2.5 MPa) used in this study. The properties ofthe HDPE and polyester fiber are shown in Table 2. 

### 2.1. The Reinforced Layers Model and Element 

**Figure 4:**
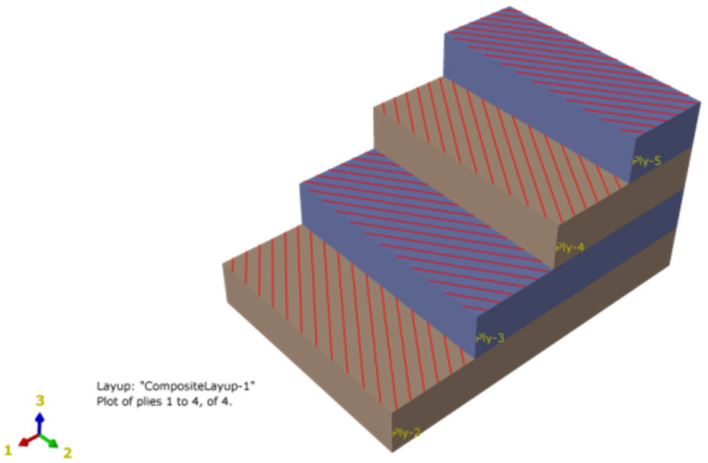
Polyester fiber layup model (4 layers).

Considering the anisotropy of reinforced polyester fiber, the composite material homogenization Halpin-Tsai model [16] was used to simulate the reinforced layer.The whole reinforced layer was regarded as a kind of orthogonal anisotropic homogeneous material that was interlaced, and four layers of polyester fibers were radially stacked from inside to outside at a winding angle of ±55°, as shown in Figure 4. The solid element C3D8R was chosen to model each layer and to complete the mesh division; 14,364 elements, 19,298 nodes, and 6 degrees of freedom for each node were assigned for the HDFE model. One layer of solid elements across a layer of material was used because the increase inlayers and finite element number had little effect on the numerical result [17]. 

### 2.2. Interaction

Surface-to-surface contact was used to simulate the interactions between each layer. The surface of the HDPE pipe was specified as the slave surface due to its softer properties compared with thoseof the polyester fiber for the reinforced layer. The normal mechanical behavior was defined as“Hard Contact” with ‘‘Allow separation after contact”, and the Penalty was taken as the friction formulation for tangential behavior. Thefriction coefficients were selected as 0.2 for the fiber-HDPE surface contact.

### 2.3. Boundary Conditions and Load

**Figure 5:**
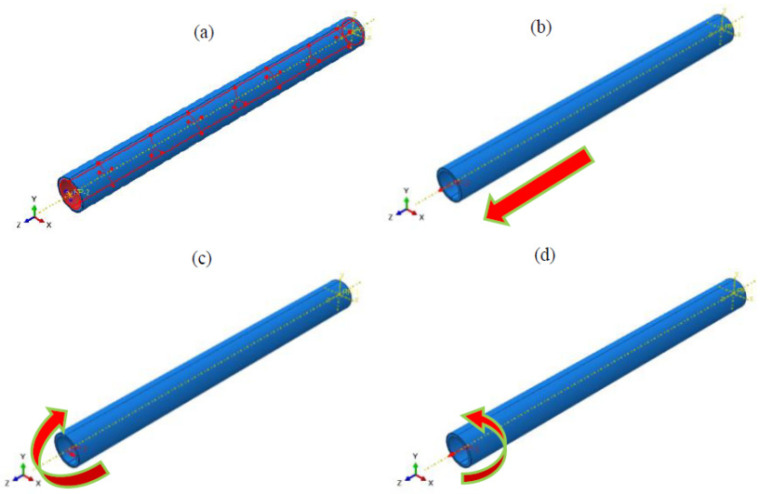
Schematicdiagram of the loading of the RTP: (**a**) inner pressure; (**b**) tension; (**c**) bending; (**d**) torsion.

The right end of the pipe was fixed, while the other was coupled to a reference point located at the center of the cross section. All of the six degrees of freedom were coupled to this point to ensure the deformation of the section was consistent and rigid. The load of inner pressure, bending, torsion, and tension was applied on the RTP, as shown in Figure 5. 

### 2.4. Verification of FEM Simulation

**Figure 6:**
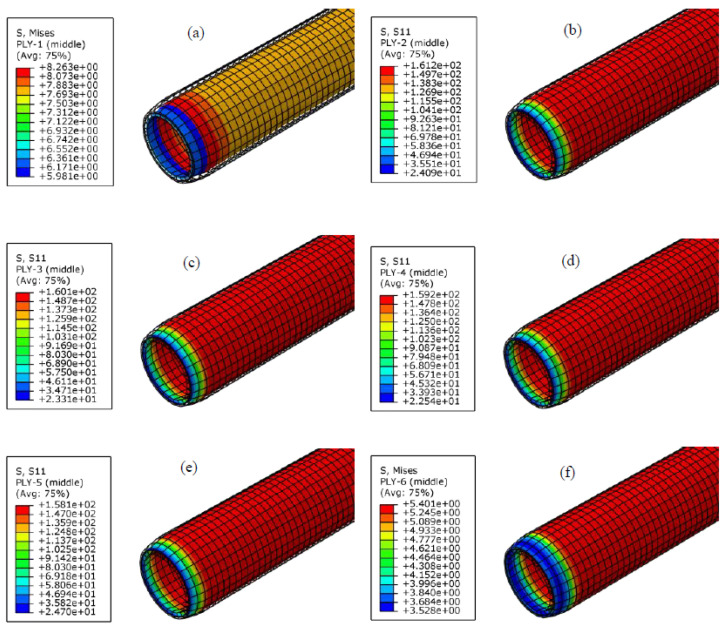
Stress distributions of each layer of the RTP at the inner pressure of 3.75 MPa: (**a**) liner; (**b**) the first reinforced layer; (**c**) the second reinforced layer; (**d**) the third reinforced layer; (**e**) the fourth reinforced layer; (**f**) outer cover.

Based on the RTP model established above, the mechanical behavior of the DN 150 polyester fiber-reinforced HDPE composite pipe under internal pressure was simulated by the finite element method. The stress distribution of each layer of the RTP under 3.75 MPa (1.5 times the nominal pressure of 2.5 MPa) was obtained, as shown in Figure 6. It can be seen that the cross-section remained circular after being subjected to the internal pressure, and the simulated outer diameter of the RTP increased by 3.17 mm. After the actual hydrostatic test (holding pressure of 3.75 MPa for 4 h) according to standard SY/T 6662.2-2012 [18], the measured outside diameter of the RTP increased by 3.24 mm. The consistency of the simulation and experimental results preliminarily showed that the established RTP model is reliable.

The stress distributions of each layer of the RTP are presented in Figure 6. All layers exhibited a similar homogeneous state of stress, and the stresses of reinforced layers were much higher than those of the inner and outerHDPE layer. It can be considered that the fiber reinforced layer is the main load bearing part of the RTP when subjected to the internal pressure. Moreover, the stress of the innermost fiber layer (the first reinforced layer in Figure 6b) wasthe largest one among the four reinforced layers. Thismeans that the first reinforced fiber layer next to the inner HDPE pipe is the most liable part to fail. As a result, the stress of the innermost fiber layer wastaken as the criterion to estimate the failure of the pipe. 

**Figure 7:**
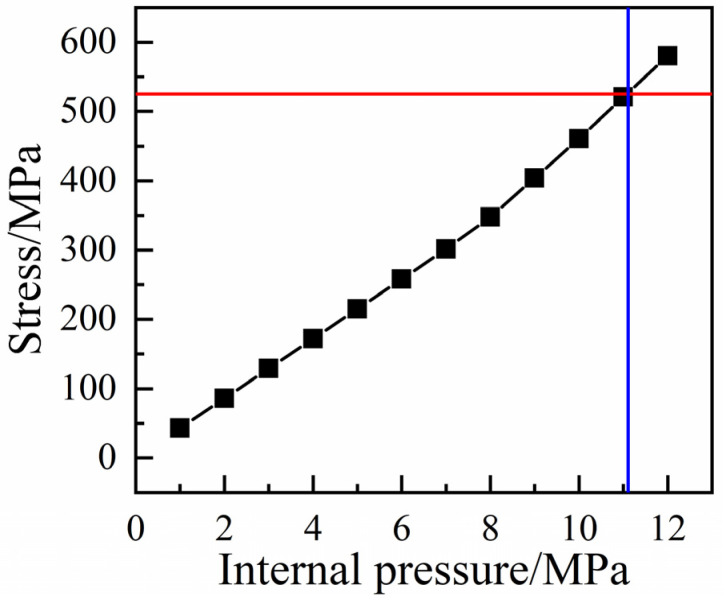
Stress-internal pressure curve for the innermost polyester fiber layer of the RTP.

In order to determine the burst pressure of the RTP, the maximum stress criterion was used as the failure criterion of the innermost polyester fiber. That is, when any of the stress components in the main direction of polyester fiber reaches its ultimate strength, the fiber would be broken, and the corresponding internal pressure is the burst pressure of the pipe. As shown in Figure 7, the stress of the polyester fiber increased with the internal pressure. When the stress value of polyester fiber reached its breaking strength of 525.2 MPa, the corresponding internal pressure (i.e., burst pressure) of the RTP was 11.105 MPa. The simulated burst pressure of 11.11 MPa is basically consistent with the experimental measured average value of 10.75 MPa, and the relative error was only 3.3%, which further confirms the availability and accuracy of the established model of the RTP.

## 3. Limit Operating Performance Analysis of RTPs

### 3.1. Tensile Analysis of the RTP

**Figure 8:**
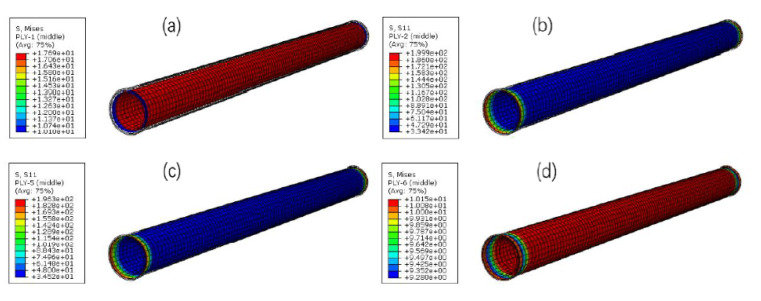
Stress distributions of each layer of the RTP at the axial tensile displacement of 100mm: (**a**) liner; (**b**) the first reinforced layer; (**c**) the fourth reinforced layer; (**d**) outer cover.

The axial displacement tension was used to study the tensile behavior of the RTP in which the incremental step of the axial displacement was 10 mm. Figure 8 shows the stress distributions of each layer of the RTP at the axial tensile displacement with 100 mm. It can be seen that the cross-section of each layer tended to shrink under the tension of the RTP. The maximum stresses of the inner liner and the outer coverwere 17.69 MPa and 10.15 MPa, respectively. There were both higher than the yield strength value 17.33 MPa and 8.66 MPa of their corresponding materials. However, the stresses of the polyester fiber in the innermost and outermost layer were 33.42 MPa and 34.52 MPa, respectively, which were much lower than their breaking strength of 525.2 MPa. Therefore, the stress of the HDPE used in the inner liner and the outer cover was taken as the criterion to estimate the tension failure of the pipe.

**Figure 9:**
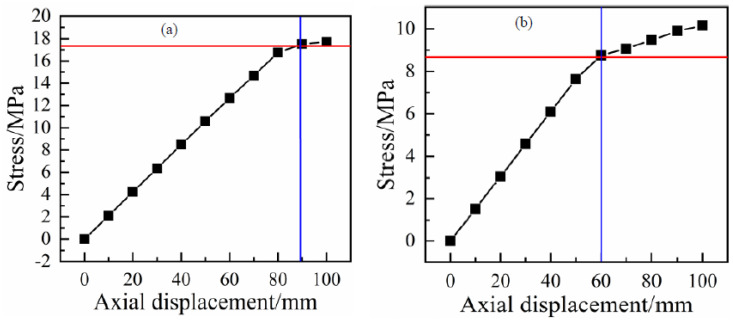
Curves of stress with axial tensile displacement: (**a**) inner liner; (**b**) outer cover.

In order to determine the criticalstrain fortension, the stresses of the inner liner and the outer cover with tensile displacement were calculated and are shown in Figure 9. It can be seen that both of the stresses for the two layers increased with the displacement. Because the tensile strength of the HDPE used for outer cover was lower than that of the inner liner, the plastic deformation first occurred at the outer cover. Accordingly, the critical axial displacement for the RTP with a length of 2000 mm was determined by the outer cover to be 60 mm(Figure 9b), and its corresponding critical strain was 3%. 

### 3.2. Flexure Analysis of RTP

The RTP being reeled onto a spool experiences large deformations. The geometrically non-linear approach was employed to simulate the mechanical behavior of the RTP under coiled bending. The Nlgeom option was chosen in the general static solution procedure of the ABAQUS/Standard to solvethe non-linear problem. Riks analysis step was used to simulate the bending process.It uses the load magnitude as an additional unknown and solves for loads and displacements simultaneously. The Riks method follows an eigen value buckling analysis to provide complete information about a structure’s failure. 

**Figure 10:**
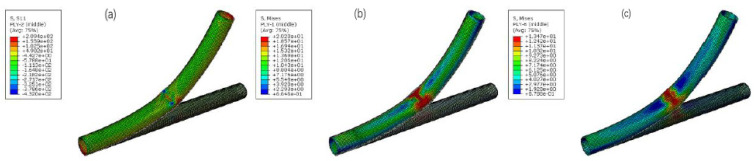
Stress distributions of each layer of the RTP at the bending load: (**a**) the innermost reinforced layer; (**b**) inner liner; (**c**) outer cover.

At the eigen value calculated by Riks, the stress distributions of each layer of the RTP are shown in Figure 10. An obvious stress concentration and buckling were observed in the middle of each layer due to bending action. The maximum stress value of the reinforced fiber layer was 209.4 MPa (Figure 10a), which was much lower than its breaking strength of 525.2 MPa. Therefore, it is difficult for the fiber layer to break even if it is dented during the bending process.However, the maximum stress values of the inner liner and the outer cover HDPE layers were 20.2 MPa and 13.47 MPa respectively, both of which werehigher than the yield strength of their corresponding material. Hence, the plastic deformation occurred at the inner liner and the outer cover layers, forming an unrecoverable depression, which led to the instability failure of the RTP. In a word, the failure criterion of bending was whether the pipe had buckling or not. 

**Figure 11:**
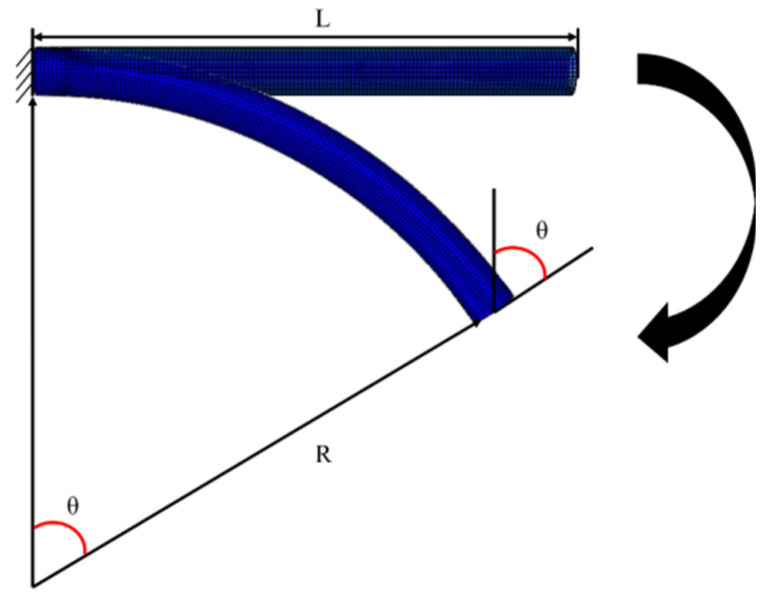
Schematicdiagram of the bending radius of the RTP.

The minimum respooling bend radius *R* isone of the key parameters of the RTP in transportation and storage process, which can be calculated by Equation (1):

*R* = *L*/*θ* (1)

where *L* is the length of the RTP (mm), *θ_c_* is the critical bending radian (rad), as shown in Figure 11. 

The critical bending radian *θ_c_* of the RTP can be obtained by FEM simulation according to above-mentioned failure criterion. In this study, the critical bending radian of the RTP (DN 150, PN 2.5 MPa) was calculated to be 1.97 rad, and the minimum respooling bend radius was calculated to be 1016.29 mm accordingly.

### 3.3. Torsion Analysis of the RTP

The static analysis step was used to simulate and analyze the torsion process of the RTP in which the torsional radian can be increased uniformly from zeroto the critical torsional load. As shown in Figure 5d, the left end of the RTP was rotated counter clockwise with the z-axis, and the torsional load was loaded in increments of 0.1 rad.

**Figure 12:**
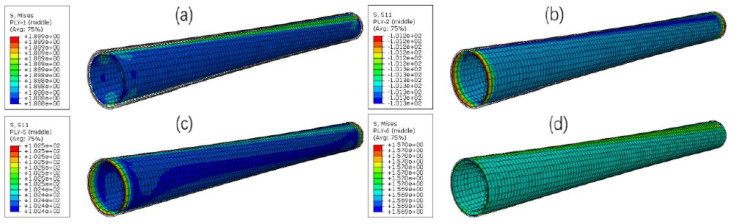
Stress distributions of each layer of the RTP at the torsion load of 0.2 rad: (**a**) liner; (**b**) the first reinforced layer; (**c**) the fourth reinforced layer; (**d**) outer cover.

**Figure 13:**
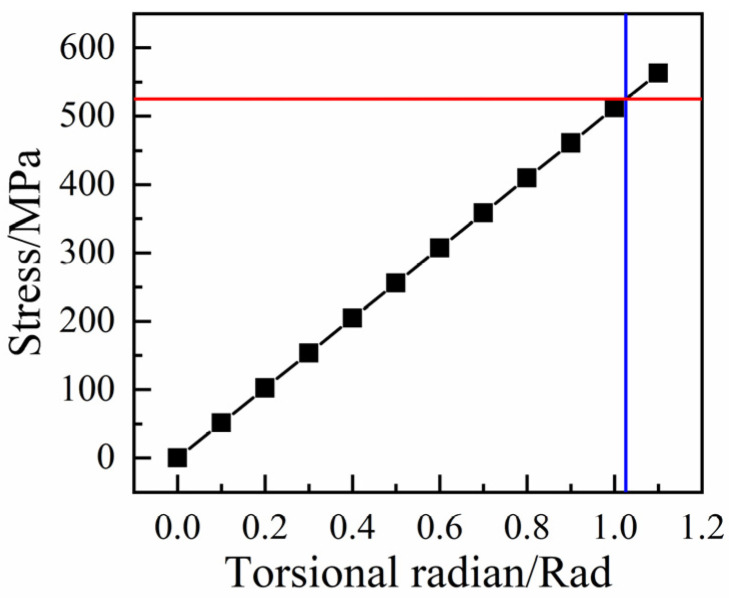
Curve of fiber stress with increasing torsional radians.

The stress distributions of the RTP under a torsion load of 0.2 rad as represented in Figure 12. The stress value of the outermost fiber was +102.4 MPa, which was slightly higher than that of the innermost fiber of −101.3 MPa (the positive and negative signs of the fiber stress value corresponding to the wound angle of ±55°). At the same time, the stress of the inner liner and the outer cover was much smaller than that of the yield strength of the corresponding HDPE material. Therefore, the polyester fiber layer was still the main bearing part of the pipe during the torsion process, and the stress of the outermost fiber layer was taken as the criterion to estimate the failure of the pipe. As the torsional radian increased, the stress of the outermost fiber increased linearly, as shown in Figure 13. When the stress of the outermost fiber reached its breaking strength of 525.2 MPa, the corresponding critical torsional radian of the RTP was 1.03 rad, and the corresponding torsional angle was 58.77°.

## 4. Experimental Verification

### 4.1. Tensile Tests

**Figure 14:**
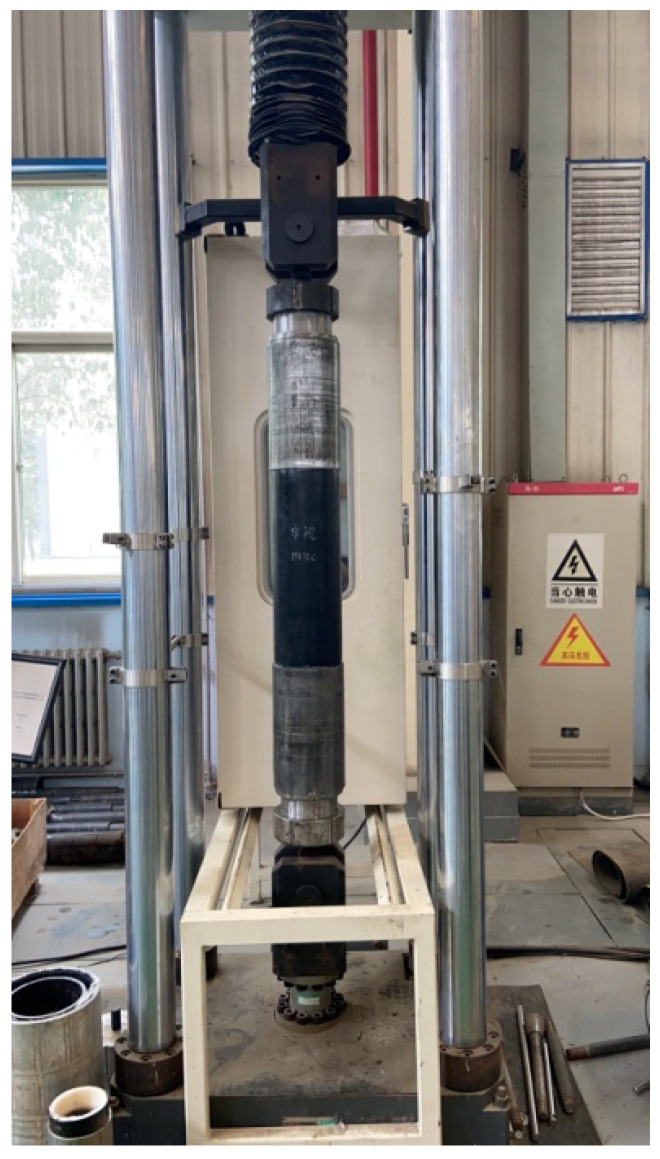
Tensile test of the RTP sample.

**Figure 15:**
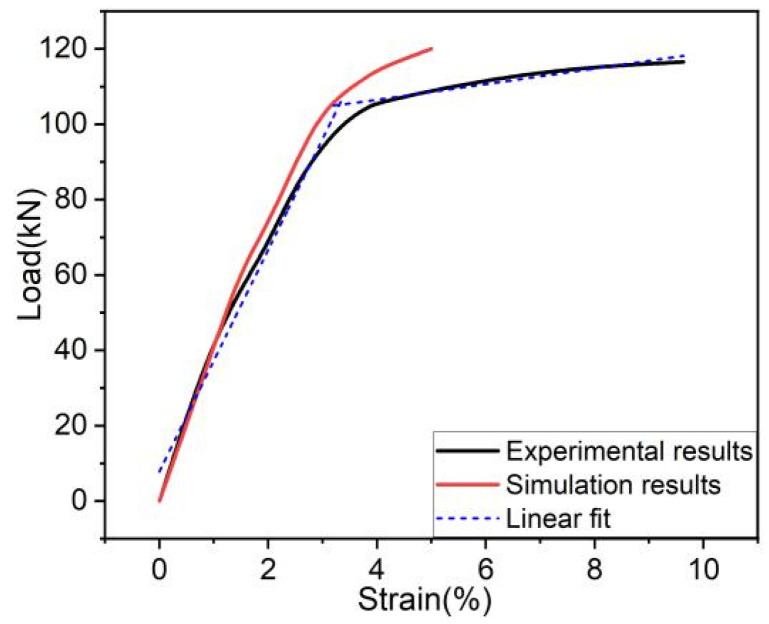
Load-strain curves obtained from the experiment and FEM.

The tensile propertyof the RTP was determined according to ASTM D2105-01(2019) [19] by using a hydraulic universal testing machine (WAW-500D-JG, SHENZHEN SUNS TECHNOLOGY STOCK CO., LTD., Shenzhen, China) with a tensile velocity of 10 mm/min, as shown in Figure 14. The length of the sample with two end fittings was 2000 mm. The comparison between the load-strain curves from the experiment and FEM is given in Figure 15. It can be seen that the curves shared the same trend in general. The maximum error of loads at the same strain between the simulation results and the experimental results was basically within 10%. Considering the simplification of the FEM model, the simulation results can be considered to be within an acceptable range. 

In practical engineering, when the tensile strain of an RTP is more than a certain value, it is considered to have failed [12]. Bai et al. reported that the strain of the HDPE layers should not exceed 7.7% for MSFP (metallic strip flexible pipes) [20]. Generally, once the stress of any layer of the RTP is higher than its yield stress, it might carry a safety risk as the pipe would lose some proportion of its stiffness during the usage. It can be noticed from Figure 15 thatthe tensile process of the RTP (DN 150, PN 2.5 MPa)consisted of a linear elastic stage and a plastic elongation stage.The critical strain is the turning point of these two stages.The determined strain values of simulation and experimental results were 3% and 3.28%, respectively. These two critical strains are very close, further implying the accuracy and reliability of the FEM model used in this study. 

### 4.2. Flexure Tests

**Figure 16:**
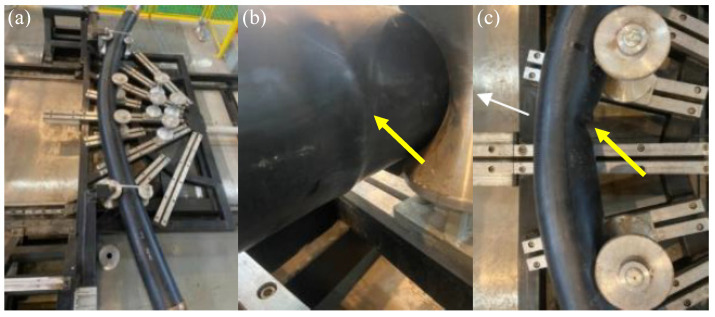
Bending test of the RTP sample. (**a**): bending radius of 2000 mm; (**b**): bending radius of 1100 mm; (**c**) bending radius of 1000 mm.

The flexure test was conducted on the Bending Tester (CHENGDE PRECISION TESTING MACHINE CO. LTD., Chengde, China). The bending properties of the RTP were tested starting with a bending radius of 2000 mm (Figure 16a)and then tested at a decreasing interval of 100 mm. Appearance of the RTP was observed by visual inspection after conditioning for four hours according to ISO 291:2008 [21] at each interval. There was an obvious morphology change (as shown in Figure 16b) when the bending radius of the RTP decreased to 1100 mm. Continued bending of the RTP to a bending radius of 1000 mm yielded an obvious buckling phenomenon that can be observed as shown in Figure 16c. It is very similar to the simulation results shown in Figure 10. Based on the experimental results, it can be concluded that the irreversible buckling failure occurs when the bending radius is less than 1100 mm. Therefore, the minimum respooling bend radius of the RTP (DN 150, PN 2.5 MPa) is 1100 mm, which is basically the same as the simulated value of 1016.29 mm.

### 4.3. Torsion Tests

**Figure 17:**
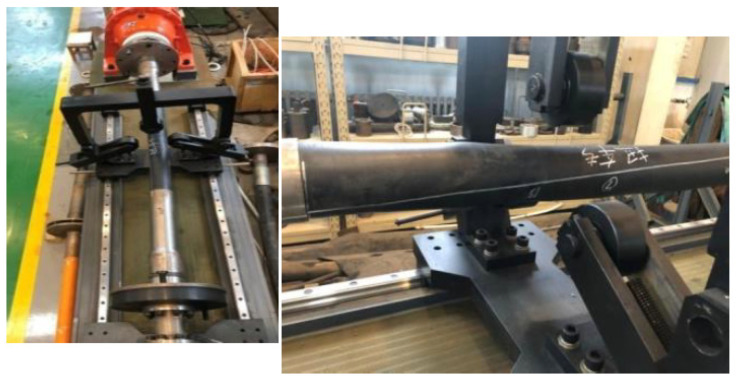
Torsion test of the RTP sample.

**Figure 18:**
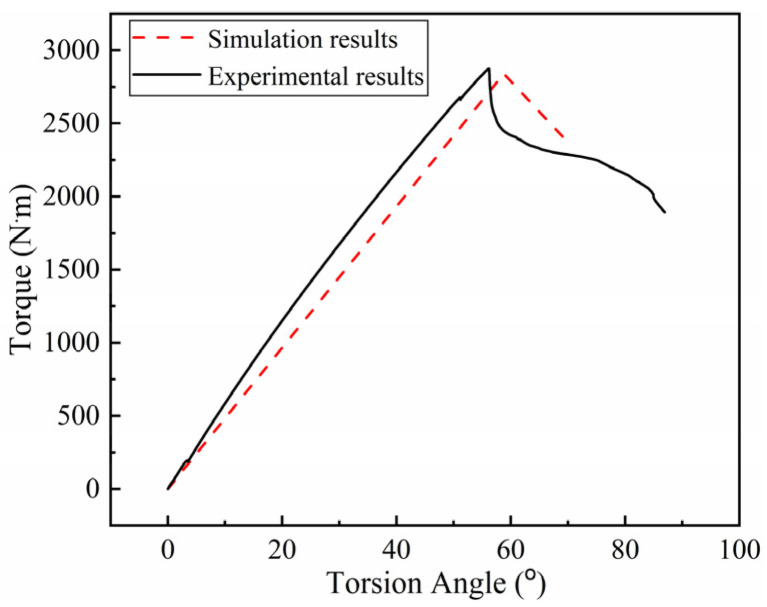
Torque–torsion angle response for the RTP.

The torsion propertyof the RTP was determined by using a Torsion Tester (Jinan Bangwei Mechanical & Electrical Equipment Co., Ltd., Jinan, China) with a velocity of 10°/min, as shown in Figure 17. The torsional behavior of the RTP is shown by the torque-torsion angle curves given in Figure 18. The torque increased linearly as the torsion angle increased. When a significant torque decline was observed on the test curve, its corresponding torsion angle was the limit torsion angle. As shown in Figure 18, the simulation curve exhibited similar characteristics to the experimental result. The simulated limit torsion angle of the RTP (DN 150, PN 2.5 MPa) was 56.17°, which is close to the experimental result of 58.77°.

## 5. Conclusions

In this paper, the finite element model of a non-bonded RTP (polyester fiber reinforced HDPE pipe, DN 150, PN 2.5 MPa) subjected to inner pressure, axial tensile, coiled bending, and torsion load was established and verified by experiments. FEM simulation determined the limit operating performances of the RTP under these different loads quickly and accurately. 

The burst pressure of the RTP obtained by simulation was 11.11 MPa, which is basically consistent with the experimental test of 10.75 MPa in the laboratory. The limit axial tensile displacement of the RTP (DN 150, PN 2.5 MPa) with a length of 2000 mm was determined to be 60 mm by simulation, and the corresponding critical strain was 3%. The critical strain obtained by the tensile test was 3.28%, indicating the accuracy and reliability of the FEM model used in this study.The minimum respooling bend radius of the RTP (DN 150, PN 2.5 MPa) was considered to be 1100 mm from the flexure test, which is basically the same as the simulated value of 1016.29 mm. The critical torsion angle of the RTP was 58.77°, similar to the experimental result of 56.17°. 

For the operating performances analyzed in this study, it is concluded that FEM is a reliable method toestablish specifications of the RTP forfield construction operation and failure prevention. However, in practical engineering, the RTP is often subjected to complicated loads such as inner pressure-tensile, inner pressure-bending, tensile-torsion, and bending-torsion, etc. The limit operating performances of the RTP will be changed and should be systemically studied further. 

## Figures and Tables

**Table 1:** The geometrical parameters of RTP.

Parameter	Value
Inner radius (mm)	148
Outer radius (mm)	180
Thickness of inner PE pipe(mm)	10
Thickness of outer PE pipe(mm)	4
Winding angle of polyester fiber (°)	+55/−55
Reinforced layer Number	4

**Table 2:** Parameters of materials used in the RTP.

Layer	Parameter	Value
Inner HDPE	Elastic modulus (MPa)	403.1
	Yield strength (MPa)	17.33
Outer HDPE	Elastic modulus (MPa)	300.8
	Yield strength (MPa)	8.66
Polyester fiber	Linear density (Dtex)	20,370
	Breaking force (N)	1650
	Breaking strength (MPa)	525.2
	Elongation at break (%)	15.2

## Data Availability

The data presented in this study are available on request from the corresponding author.
